# Intact neurophysiological markers of death denial in long-term ayahuasca users

**DOI:** 10.1007/s00213-025-06963-z

**Published:** 2025-11-10

**Authors:** Yair Dor-Ziderman, Jonathan David, Aviva Berkovich-Ohana

**Affiliations:** 1 Integrated Brain and Behavior Research Center (IBBRC), University of Haifa, Mount Carmel, Haifa, 3498838 Israel; 2 Edmond J. Safra Brain Research Centre, University of Haifa, Haifa, Israel; 3 School of Therapy, Counseling, and Human Development, Faculty of Education, University of Haifa, Haifa, Israel

**Keywords:** Ayahuasca, Psychedelics, Fear of death, Death acceptance, Self, Magnetoencephalogram (MEG), Visual mismatch response (vMMR)

## Abstract

**Rationale:**

There is growing enthusiasm around the potential of psychedelics to transform how individuals engage with the theme of death, with self-report evidence suggesting these substances foster acceptance and reduce fear-of-death. Yet, given extensive evidence linking unconscious processes to mortality avoidance, current research efforts remain limited and call for implicit and neurobiology-based measures.

**Objectives & methods:**

To this end, we applied a validated no-report magnetoencephalogram (MEG) visual MisMatch Response (vMMR) paradigm to assess whether long-term ayahuasca users (*N* = 50), compared to previously published data from healthy controls and experienced meditators, show self-specific neurophysiological markers of death-denial/acceptance. Self-report and behavioral measures of fear-of-death were also collected.

**Results:**

Cluster-based permutation analyses on the vMMR data showed that ayahuasca users’ brains responded to the coupling of death and self-stimuli in a manner indicating denial rather than acceptance. At the same time, 1-way ANOVAs and *post hoc t*-tests analyses indicated that the ayahuasca group evidenced less implicit fear-of-death than the general population, and less explicit fear-of-death than both the general and meditator samples. Further correlation analyses on the neurophysiological ayahuasca sample death-denial marker supported its construct validity by associating it with less self-reported death acceptance and reduced accessibility to death-related thoughts. Finally, death-denial was linked to greater life satisfaction, supporting its adaptive role.

**Conclusions:**

These findings provide preliminary evidence that while ayahuasca may alter how humans interact with the theme of death on conceptual and affective levels of cognitive processing, on automatic, perceptual unconscious levels of self-specific processing, death-denial processes appear to remain intact.

**Supplementary Information:**

The online version contains supplementary material available at https://doi.org/10.1007/s00213-025-06963-z.

## Introduction

Death is an inescapable reality. Across different cultures and throughout history, humanity has developed a variety of strategies to cope with the existential anxiety that arises from the awareness of life’s finality. These strategies include, among others, adopting cultural norms, endorsing religious beliefs, partaking in philosophical discourses, engaging in contemplative practices, and consuming psychoactive substances within ritualistic settings.

### The impact of psychedelics on death processing

In recent years, there has been a resurgence of interest in the potential therapeutic benefits of 5-HT2A agonists, also known as classic psychedelics (Nichols 2016; Hadar et al. 2022). One of the most enthusiastic areas where psychedelic medicine has gained traction is the belief that these substances can transform our relationship with one of the most profound and complex human existential themes – death. Beginning in the 1960 s (Kurland et al. 1972; Grof and Halifax 1977) and experiencing a resurgence in the current psychedelic renaissance, initial evidence from both naturalistic (David et al. 2025; Davis et al. 2020; Griffiths et al. 2019; Moreton et al. 2023a, b, 2024; Sweeney et al. 2022; Yaden et al. 2017) and clinical settings (Gasser et al. 2015; Griffiths et al. 2016, 2018) suggests that psychedelics may promote a greater acceptance of death and reduce fear and anxiety of death. Several mechanisms have been proposed (David et al. 2025; Letheby 2024; Moreton et al. 2020), and it has been even suggested that the death anxiety-reducing effects of psychedelics may serve as a mediator of their general therapeutic benefits (Moreton et al. 2020).

This growing body of research has contributed to a surge in scientific, clinical, and public interest, as well as extensive media coverage. From a scientific and clinical perspective, psychedelics have been proposed as a potential treatment option for end-of-life care-related anxiety (Reiche et al. 2018), with more than 20 registered trials to date investigating this possibility (Jing et al. 2023). This momentum has also been embraced by governmental funding, as evidenced by a recent award of over €6.5 million by the European Union to study psychedelics for treating psychological distress in palliative care (Payne 2024). The public interest and media coverage surrounding this topic have fueled an even greater hype. As Letheby (2024, p. 2) notes:

“A glance at recent headlines could lead one to the conclusion that psychedelics reduce fear of death as reliably as aspirin reduces headaches. Here is a sample: ‘Hallucinogenic drugs help cancer patients deal with their fear of death’ – Science magazine, ‘Facing Death Without Fear: Psychedelics for End-of-Life Care’ – WebMD, ‘Psilocybin: A Journey Beyond the Fear of Death?’ – Scientific American, ‘Curing the Fear of Death: How “Tripping Out” Could Change Everything’ – Salon, and ‘Taking Psychedelics Could Make People Less Afraid of Dying’ – Time magazine.”

Despite the emerging research and surrounding hype, the body of evidence that remains is still limited. In addition to rigorous studies failing to demonstrate any effect of psychedelics on death anxiety (Ross et al. 2016), most of the relevant research has relied on online surveys (Davis et al. 2020; Moreton et al. 2024; Moreton et al. 2023a, b; Sweeney et al. 2022; Yaden et al. 2017; Griffiths et al. 2019), which have significant limitations (Buchanan 2018). Some of these studies have relied on a single question, such as ‘Did psychedelics change your perception of death?’ (Davis et al. 2020; Yaden et al. 2017; Griffiths et al. 2019). Most importantly, nearly all research in this area, including well-designed clinical studies (Griffiths et al. 2016, 2018), depend solely on self-reports and explicit measures. This issue becomes particularly crucial in the context of studying death-related aspects of human experience, considering the hundreds of studies grounded in the Terror Management Theory (TMT) paradigm (Greenberg et al. 1986; Pyszczynski et al. 2015) documenting a wide array of defensive processes which manage existential terror by reinforcing self-esteem, adhering to cultural worldviews, and solidifying group identities (Burke et al. 2010). Crucially, these processes unfold below the threshold of conscious awareness. Thus, any account of the impact of psychedelics on death processing would be incomplete without data stemming from implicit, behavioral, and no-report neurobiologically-informed measures.

While the neurobiological mechanisms underlying psychedelic-induced changes are not fully understood, several theories attempting to explain how these substances affect the brain have emerged (Doss et al. 2021). Arguably, the most widely accepted brain brain-driven theory on the mechanism of psychedelics is the Relaxed Beliefs Under Psychedelics (REBUS) theory (Carhart-Harris and Friston 2019). An important advantage of REBUS is that it is anchored in the predictive coding framework, which is a general theory of brain function with a robust computational scope. Predictive processing conceptualizes the brain as a prediction machine functioning as a hierarchical generative model of the world, guided by Bayesian probability principles (Friston 2005; Hohwy 2013; Clark 2013). Within this framework, the brain is viewed as a statistical organ, constantly striving to make sense of a world concealed from direct perception by generating simulations (or statistical models) of its internal and external environments. These models are continuously refined and adjusted through sensory feedback. When there is a mismatch between expected and actual sensory input—referred to as ‘prediction errors’—the brain either updates its predictions to better align with the sensory data or by interacting with the environment to confirm its expectations. The process of modifying top-down predictions (or prior beliefs) to incorporate bottom-up prediction errors involves a complex interplay between descending and ascending neuronal signals (Bastos et al. 2012). This process is further refined through ‘precision weighting,’ where the brain modulates the influence of these signals based on their estimated reliability (Friston 2005). According to the REBUS model, psychedelics exert their acute effects by relaxing rigid, high-level prior beliefs (i.e., top-down signals), which are typically resistant to change. This relaxation allows new bottom-up information to reach consciousness, creating a state in which individuals’ brains can reassess and reorganize their cognitive frameworks. This process may lead to long-term changes in behavior, perception, and thought patterns (Carhart-Harris and Friston 2019). Recently, the REBUS model has been proposed as a computational neurobiological framework for the long-term reduction in death anxiety following psychedelic use (Letheby 2024). However, no studies have yet investigated this acutely or as a long-term effect.

### The death denial visual mismatch response paradigm

The paradigm introduced in Dor-Ziderman et al. (2019) is a self-specific (processes differentiating self from other (Christoff et al. 2011) prediction-based neuroscience paradigm. Death-denial was defined as an encoded statistical prior belief that ‘death concerns others, but not our self’, and was operationalized via a fully randomized 2 × 2 within-subjects MEG visual MisMatch Response (vMMR (Kremláček et al. 2016) paradigm using *self* and *other* facial images (Sel et al. 2016) coupled with either *death* or merely *negative* linguistic stimuli (see Methods section and SI1 section for more details on this task).

**Fig. 1:**
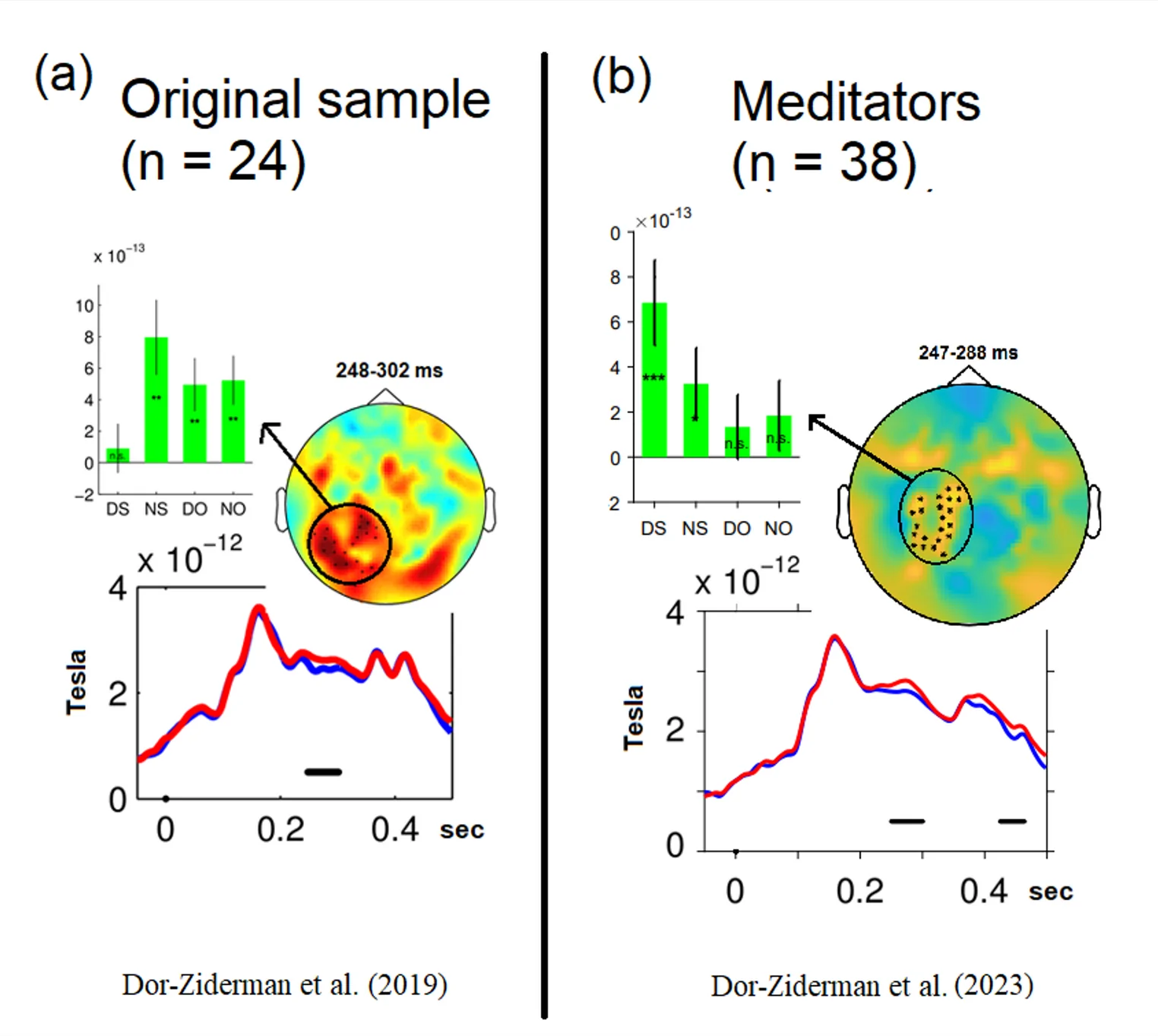
vMMR sensor-level results from previous studies. Summarized results from (**a**) (Dor-Ziderman et al. 2019) showing null results for (and only for) the DS condition, and from (**b**) (Dor-Ziderman et al. 2025) showing a main effect (self > other) beyond death or negative linguistic context. Both subfigures display significant time-window (bottom), significant 2D maps of sensor clusters (top-right), and vMMR difference plots (top-left). Error bars denote standard error of the mean. *=*p* <.05, **=*p* <.01, ***=*p* <.001

Using this paradigm, we reported (Dor-Ziderman et al. 2019) (Fig. 1a) vMMR effects at ~ 250–300 ms post deviant stimuli presentation over posterior-left sensors under a negative context regardless of face identity (negative-self and negative-other conditions, NS and NO respectively) and under a death context when a stranger’s facial image was presented (death-other condition, DO). Importantly, the presentation of *self* images displayed under a death-related word (DS condition) resulted in a sharp attenuation of this basic predictive perceptual mechanism as indicated by a lack of a detectable vMMR effect. These results, which indicated a ‘self-disadvantage’ under existential threat (the attenuation of DS vMMN), led to the construction of the Death Self-Disadvantage (DSD) index, computed by subtracting the DS from the DO condition (as well as control Negativity Self Disadvantage (NSD) index computed by subtracting the NS from the NO condition). The DSD indexes a self-specific prediction-based neurophysiological marker of death denial, and can be interpreted as a shielding of the self model from information which could undermine its coherency (Metzinger 2024). Importantly, the DSD was positively correlated with a behavioral measure related to fear of death (see Methods section), suggesting that increased cognitive load for processing self-relevant death-related information was associated with the neurophysiology of death denial.

A follow up study using the same paradigm was conducted on a cohort (*n* = 38) of experienced meditators (Dor-Ziderman et al. 2025). The results showed that the meditators’ brains were not aversive to the combination of death and self-related information (there was a significant DS vMMR effect) (Fig. 1b). Rather, the results indicated the well-known self-advantage effect in a manner that did not differentiate between death- or negative-related conditions. These results were interpreted as indicating acceptance rather than denial of death. Finally, the study also presented correlational evidence regarding a link between degree of neurophysiologically-measured death acceptance and positively-valanced meditation-induced self-dissolution experiences (assessed via quantified phenomenological invariants, see (Nave et al. 2021). Overall, the study presented compelling evidence that the neural mechanisms underlying the human automatic tendency to avoid death were not hard-wired but were rather amenable to change, possibly in association with self-dissolution experiences.

### Study aims and hypotheses

In the present study we aimed at filling the evidence gap regarding the impact of psychedelics, and specifically ayahuasca, on long-term unconscious neurobiologically-informed metrics of death processing. We focused on experienced users of ayahuasca (*n* = 50), also known as ‘the vine of death,’ a potent indigenous Amazonian psychedelic decoction containing mainly N, N-Dimethyltryptamine (DMT) and harmala alkaloids (Shanon 2005). This focus was motivated by the brew’s unique association with the theme of death (Shanon 2005; David et al. 2023, 2025). Employing the validated prediction-based MEG vMMR paradigm and building on the reviewed literature, we hypothesized [H1] that experienced ayahuasca users’ vMMR data would display a significant response to deviancy in the condition coupling death-related words and self faces, in a manner similar to the meditators, and unlike the data reported for the original general population sample.

We also took advantage of the study’s relatively large ayahuasca sample size to perform a number of exploratory analyses related to the construct validity of the vMMR paradigm, its possible adaptive role, and link to consciously experienced affect. More specifically, we [H2a] examined the construct validity of the DSD, that is, whether other measures indexing a global conceptual representation of death acceptance (vs. denial), or cognitive processes related to denial of death-related thoughts would be associated with it. A second analysis targeted the role of death denial as a defense mechanism, acting as a protective ‘shield’ from existential fear and anxiety (Pyszczynski et al. 2015; Baillie 2019). We thus [H2b] examined whether the DSD would be associated with self-reported death anxiety and fear of death, as well as with the behavioral measures of the latter, which was shown in a previous study (Dor-Ziderman et al. 2019) to correlate with this measure. Finally, due to the hypothesized adaptive value of death defenses (Pyszczynski et al. 2015; Qirko 2017; Varki 2019), we [H2c] explored whether the presence of death denial mechanisms impacted life in a positive way. We looked at a measure of psychological well-being, shown in a previous study to be correlated with death acceptance (Dor-Ziderman et al. 2025), as well as a measure examining a global assessment of perceived life satisfaction.

## Methods

### Participants

The sample consisted of healthy adults (*n* = 50, 17 female; age = 38.4 ± 8.93 ranging from 25 to 68 years) recruited via social media and personal connections. An a priori sample size justification was conducted based on projected effect sizes conservatively derived from (Dor-Ziderman et al. 2019). Using G*Power 3.1, we estimated that a medium effect size (f = 0.25) with desired power = 0.95 at α = 0.05 for detecting within-subject vMMR effects would require *n* = 36. Thus, our final sample (*n* = 50) was adequate. Inclusion criteria were willingness to sign the informed consent, multiple ayahuasca usage (≥ 8 times), consideration of ayahuasca as one’s primary psychedelic substance-of-use, and normal or corrected-to-normal vision. Mean lifetime use (number of ceremonies in which ayahuasca was consumed) was 58.4 ± 84.8. Exclusion criteria were conditions limiting MEG data quality (dental splints, artificial cardiac pacemakers), current use of psychoactive medications (antidepressants, mood stabilizers, anxiolytics, and antipsychotics), use of drugs of abuse (e.g. cocaine), cannabis consumption of ≥ twice a month, neurological or active psychiatric illnesses (e.g., epilepsy, depression), loss of a first degree relative (spouse, parent, child, or sibling) within the last 12 months, and consumption of ayahuasca or other psychedelics in the 28 days preceding the assessment (for avoiding afterglow effects (Evens et al. 2023). For more information regarding demographics and ayahuasca usage habits see SI Table 2. The study was approved by the Institutional Review Board of the Faculty of Education, University of Haifa, Israel (approval number 228/21). Participants gave their written consent and were financially compensated for their time.

### Measures

#### vMMR death denial task

**Fig. 2:**
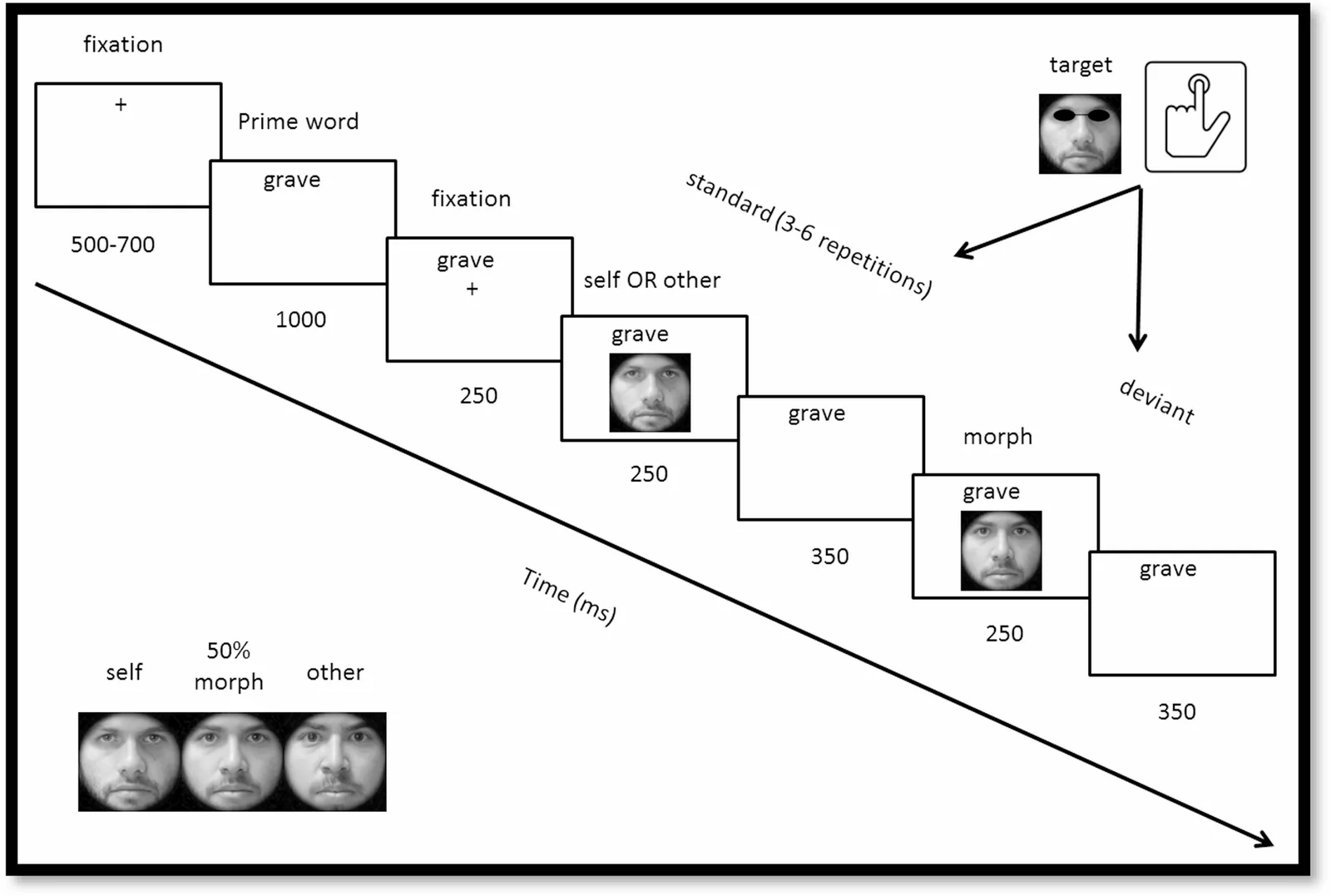
Setup of the vMMR experiment. Time course and trial stimuli. Participants were shown a (death-related or negative) prime word for 600ms. After that, underneath the word, 3–6 repetitions of standard (either self or other faces) and then a deviant face (50% self-other morphs) were shown. Faces were shown for 250ms followed by a blank image for 350 ms. To maintain alertness and focus on the facial stimuli, participants were tasked with pressing a button when a target stimuli (face with sunglasses) was detected. There were 360 total trials delivered in 3 blocks. 90 target trials (randomly appearing), 4 conditions (fully randomized) averaging 67.5 trials per condition, 1:4.5 standard to deviant ratio. Figure borrowed from (Dor-Ziderman et al. 2019)

The vMMR task’s facial stimuli, word stimuli, and procedure were similar to those employed in our previous publication (Dor-Ziderman et al. 2019, 2025). These are described briefly below. For more details regarding the facial and verbal stimuli, as well as the procedure, see supplementary section SI1: vMMR task Stimuli as and the procedure section. As illustrated in Fig. 2, a fully-randomized 2 × 2 design was implemented using PRIME (DEATH or NEGATIVE) and IDENTITY (SELF or OTHER) as within-subject factors. vMMR responses were obtained by presenting deviant facial stimuli (self-other morphed images) following 3–6 presentations of standard stimuli consisting, in each trial, of either *self* (the participant’s) or *other* (a stranger’s) facial images. Prior to the presentation of these facial sequences, a word appeared above the center of the screen to provide a context which was either death-related or merely-negative. This word remained for the duration of the sequence. Word categories were balanced for length, valence, arousal, and frequency. At any point during the sequence, a target face could appear matching the sequence type, and subjects were instructed to press a button with their index finger as soon as it was detected. Hit rates as well as response latencies were collected. Beyond maintenance of participants’ alertness and focus on the facial stimuli, these behavioral measures were included to rule out attentional factors.

#### Self-report and behavioral measures

A number of further self-report and behavioral data were collected in the current study for examining links between the neurophysiological death denial index (the DSD) and (H2a) denial/acceptance processes (death-thought suppression and death acceptance), (H2b) death-related affective (fear and anxiety of death). Each of these constructs was probed by both an explicit self-report measure, as well as a behavioral implicit measure, with the former serving to align results with the existing literature on fear/anxiety of death and the latter to explore behavioral implicit measures which are large remained unexplored (see Table 2 below). Additionally, (H2c) the relation between life flourishing and the vMMR index were also explored (well-being and satisfaction with life). Finally, (d) ontological afterlife beliefs, ayahuasca usage habits and related ego dissolution experiences were also probed and ruled out as intervening factors. Importantly, the fear-of-death measures were previously employed (Dor-Ziderman et al. 2019, 2025), thus allowing comparison with the previous healthy controls and meditators samples. Group differences were assessed via 1-way ANOVAS with post hoc dependent-samples t tests, and correlations were conducted using Pearson or Spearman analyses, depending on the measures’ data distribution (assessed via Shapiro-Wilk’s *W*).

**Table 2:** DSD correlations with explicit questionnaires and implicit behavioral measures of death-related affect, and denial, as well as with questionnaires measuring and life flourishing

	Measure	Type (explicit/implicit)	*n*	Correlation with DSD (*r*, *p*, 95% CI)
Affect	Death anxiety scale (DAS)	explicit	50	*r* =.15, *p* =.29, [−0.13 0.41]
	Fear of death (FPDS_P)	explicit	49	r_s_=−0.1, *p* =.47
	Fear of death (FPDS_RT)	implicit	49	*r*=-.09, *p* =.55, [−0.36 0.2]
Denial	Death acceptance (LAPR-DA)	explicit	50	***r=-.3***, *p* **=.03,[−0.53 − 0.02]**
	Death thought accessibility (DTA)	implicit	49	**r**_**s**_**=−0.278**, ***p*** **=.053**
Life flourishing	Life satisfaction (SWL)	explicit	50	***r*** **=.36**, ***p*** **=.01**,** [0.1 0.58]**
	Well-being (WB)	explicit	50	*r*=-.13, *p* =.37*,* [−0.39 0.15]

#### Death-related affective processes

*Death Anxiety scale* (DAS (Templer 1970) is the gold-standard self-report measure of death anxiety. The scale has been extensively used and validated in numerous languages over hundreds of studies. Its items include 15 questions such as “I am very much afraid to die.” With which participants signify their agreement using a 5-point Likert scale. Higher values denote higher death anxiety.

*Fear of death* was gauged via the Fear of Personal Death Scale (FPDS, Florian and Kravetz 1983). The FPDS is a widely used self-report scale measuring fear of death. It consists of 31 questions rated on a 7-point Likert scale, where respondents express their level of fear by responding to statements such as ‘I am afraid of death because…’”. In the present study, a modified version of the FPDS (as in (Dor-Ziderman et al. 2019; David et al. 2025) was used, where the 31 original items were presented as a forced-choice yes-no computerized questionnaire. This presentation method allowed deriving an explicit and implicit measure relating to fear of death. The explicit measure was computed as the percentage of ‘yes’ responses (FPDS_P), with higher scores indicating a greater explicit fear of death. The implicit measure (FPDS_RT) was computed by averaging the reaction times (RTs) associated with these responses. To reduce interpersonal variability, the RTs were normalized by subtracting from them the latencies of a control (equally-long) 31-items fear of dental pain questionnaire (the most commonly used control condition for mortality salience manipulations in the TMT literature (Burke et al. 2010). Longer processing times on the FPDS_RT are interpreted as reflecting greater emotional load, which is partially justified by longer latencies of death-related questions relative to dental pain-related questions (Dor-Ziderman et al. 2019; David et al. 2025).

#### Death denial/acceptance processes

*Death acceptance* was gauged via The Life Attitude Profile Revised Death Acceptance sub-scale (LAPR-DA, Reker 1992). LAPR-DA is a reliable and valid 8-item measure of explicit death acceptance. Each item is rated on a 7-point scale, ranging from 1 (“strongly disagree”), to 7 (“strongly agree”), with higher scores on this measure indicating greater explicit death acceptance.

*Death Thought Accessibility* (DTA (Greenberg et al. 1994) is a central widely used and validated TMT measure gauging the level of death thoughts activation shown to be associated with both proximal and distal defenses (Hayes et al. 2010). The Hebrew DTA version, used in numerous previous studies (e.g. (Mikulincer and Florian 2000; Florian et al. 2002)) consists of 20 word fragments, 10 of which can be completed with a death-related or neutral word (for example COFF_ _ completed as coffin or coffee). Participants were instructed to jot down the first word which came to their mind. Higher DTA values, measures as the sum of word fragments completed as death-related words, indicate higher accessibility of death-related thought. Accessibility is interpreted here as a measure of implicit death (thoughts) acceptance.

#### Life flourishing

*Well-being* (WB) was measured using the General Well-Being Scale (GWBS, (Dupuy 1978). The GWBS is a validated 18-item questionnaire designed to assess overall well-being. The first 14 items use a 6-point Likert scale, while items 15 through 18 use a 10-point Likert scale. An example question is: “How happy, satisfied, or pleased have you been with your personal life? (During the past month).” The final score is the average of all item scores, with higher values indicating greater well-being.

*Life satisfaction* (SWS) was measured via the Satisfaction With Life Scale (SWLS, (Diener et al. 1985). The SWLS is a 5-item questionnaire designed to assess an individual’s overall life satisfaction. Participants respond to statements such as “At the present time, I am satisfied with my life” on a 7-point Likert scale, where higher values indicate greater life satisfaction.

#### Additional self-report measures

The values of the measures described in this section are all reported in the supplementary information, SI Table 2.

*Demographic* information including age, gender, education, family status, income, and religion.

*Afterlife belief certainty (ABC)* was measured via a novel tool (introduced in (Dor-Ziderman et al. 2025) where participants responded on a numerical scale ranging from − 10 to 10, with − 10 indicating certainty in death as annihilation (“when the heart and brain stop working the soul/consciousness terminally ends”) to 0 (“I don’t know”) to 10 indicating certainty in continuation after death (“The soul/consciousness continues after death”).

*Ayahuasca usage habits* including life time usage, age of first ceremony, time since most recent, and time since most intense ceremony were collected.

*Self/ego-dissolution experiences* occurring during ayahuasca ceremonies were retrospectively measured via the Ego Dissolution Inventory (EDI) (Nour et al. 2016). The EDI is an 8-item self-report measure designed to capture past ego dissolution experiences. Each item in the inventory is rated using a visual analog scale format (0–100), where zero represents “No, not more than usual,” and 100 represents “Yes, entirely or completely.” Following the approach outlined in the original article by Nour et al. (2016), we used the EDI to assess both the “most intense” as well as “typical” past ego dissolution experience.

### MEG data collection, preprocessing, analyses and statistics

MEG data collection, preprocessing, analyses and statistics were similar to that employed in our previous publication (Dor-Ziderman et al. 2019). These are described briefly below.

#### Data collection

Continuous brain magnetic activity was measured using a whole-head 248-channel magnetometer array (4-D Neuroimaging, Magnes 3600 WH) while participants lay in a supine position inside a magnetically shielded room. The data were sampled at 1017.23 Hz, with an online band-pass filter ranging from 0.1 to 400 Hz. Environmental noise was mitigated by reference coils placed above the head along the x, y, and z axes. Five coils were affixed to the participant’s scalp to determine head position relative to the 248-sensor array. The head shape was captured manually using a Polhemus Fastrak digitizer. A photosensitive diode on the screen registered the onset timing of visual stimuli, while manual responses were collected using a response box.

#### Data preprocessing

The data were processed using the FieldTrip toolbox (Oostenveld et al. 2011) in conjunction with custom analysis scripts developed in MATLAB R2020a (MathWorks, Natick, MA, USA). External noise sources, such as power-line interference, mechanical vibrations, SQUID electronics-related jumps in the MEG signal, and heartbeat artifacts, were eliminated using a predefined algorithm (Tal and Abeles 2013). Two malfunctioning channels were identified and excluded from subsequent analyses. The data were divided into 600 ms epochs, starting 100 ms before and ending 500 ms after the face presentation, ensuring the longest possible epochs without overlap between trials. These segments were screened for artifacts using a semi-automated procedure. A high-pass filter of 60 Hz was applied to identify and reject trials with muscle artifacts. Subsequently, independent component analysis (Bell and Sejnowski 1995) was employed to eliminate residual variance associated with eye blinks, eye movements, and heartbeat artifacts (Jung et al. 2000). A final visual inspection was conducted to remove any remaining artifact-contaminated trials. Final mean (± std) number of trials per condition were 62.2(± 7.6), 61.1(± 8.59), 62.3(± 8/89), 63.2(± 7.61), for the DS, DO, NS, NO conditions, respectively. Trial counts were not different between conditions as indicated by a repeated-measures ANOVA (F(1,49) = 0.66, *p* =.42, *n.s.*).

*Event-related fields* (ERFs) were computed by initially applying a two-pass Butterworth filter with a fourth-order filter and a 40 Hz cutoff frequency for low-pass filtering the data. Baseline correction was performed using a 100 ms interval preceding the presentation of faces. Subsequently, a planar gradient transformation was applied (Bastiaansen and Knösche 2000), which facilitates the interpretation of sensor-level data by typically aligning the peak signal directly above the source (Hämäläinen et al. 1993). To prevent biased results, we did not use the grand average data to manually select time windows and electrode sites, following evidence that this practice can produce misleading findings (Luck and Gaspelin 2017). Determination of the relevant time points and sensors was carried out using non-parametric cluster-based permutation tests (details in the statistics section) through paired-samples *t*-tests between standard and deviant trials. These tests were first applied to the time domain (averaged across all conditions, sensors, and trials) and then to the sensor domain (averaged across the specified time window, conditions, and trials). To avoid circular analysis (Kriegeskorte et al. 2009), we employed orthogonal contrasts (Litvak et al. 2011; Kilner 2013; Luck and Gaspelin 2017) for both data selection (identifying times and sensors of interest) and hypothesis testing (comparing the different conditions). Additionally, trials were balanced for each condition within each participant before averaging to ensure unbiased selection and avoid inflating type I errors (Brooks et al. 2017). We also ensured by applying independent-sample *t* tests that the overall number of trials in the ayahuasca dataset (222 ± 30.2) was not different from the meditators (212 ± 37.5; *t* = 1.44, *p* =.155, *n.s.*), and from the general population (233 ± 15.5; *t*=−1. 6, *p* =.114, *n.s.*), samples. Finally, for each participant, ERF power values were averaged across the significant sensor cluster and time window, and difference waveforms (deviant minus standard trials) were generated. These difference waveforms were analyzed using repeated-measures and mixed-design ANOVAs, with post-hoc one-sample, paired-sample, and independent-sample *t*-tests, all corrected for multiple comparisons using the Holm-Bonferroni (*p*_*HB*_) procedure (Holm 1979).

#### Nonparametric cluster-based permutation statistics

To identify significant spatial clusters of differential ERF activity, nonparametric cluster-based permutation statistics (Maris and Oostenveld 2007) were conducted on both the temporal and spatial domains. This type of testing controls the type I error rate in multiple comparisons by detecting clusters of significant differences across space/time. This method was applied at both the sensor and source levels, chosen for its non-reliance on assumptions about the underlying distribution and its robustness against partial dependencies between neighboring sensors or voxels. Moreover, this approach has been shown to maintain nominal false-positive rates for spatial extent (Eklund et al. 2016). Cluster-level statistics, calculated as the sum of *t*-values within each cluster, were evaluated against a permutation distribution of the maximum (or minimum) cluster-level statistic. This distribution was estimated by performing 1,000 random permutations of the observed data. The resulting p-values indicate the probability, under the null hypothesis (no difference between conditions), of obtaining a maximum (or minimum) cluster-level statistic that exceeds the observed cluster-level statistics.

## Results

### Ayahuasca long-term users evidence neurophysiological indices of death denial

**Fig. 3:**
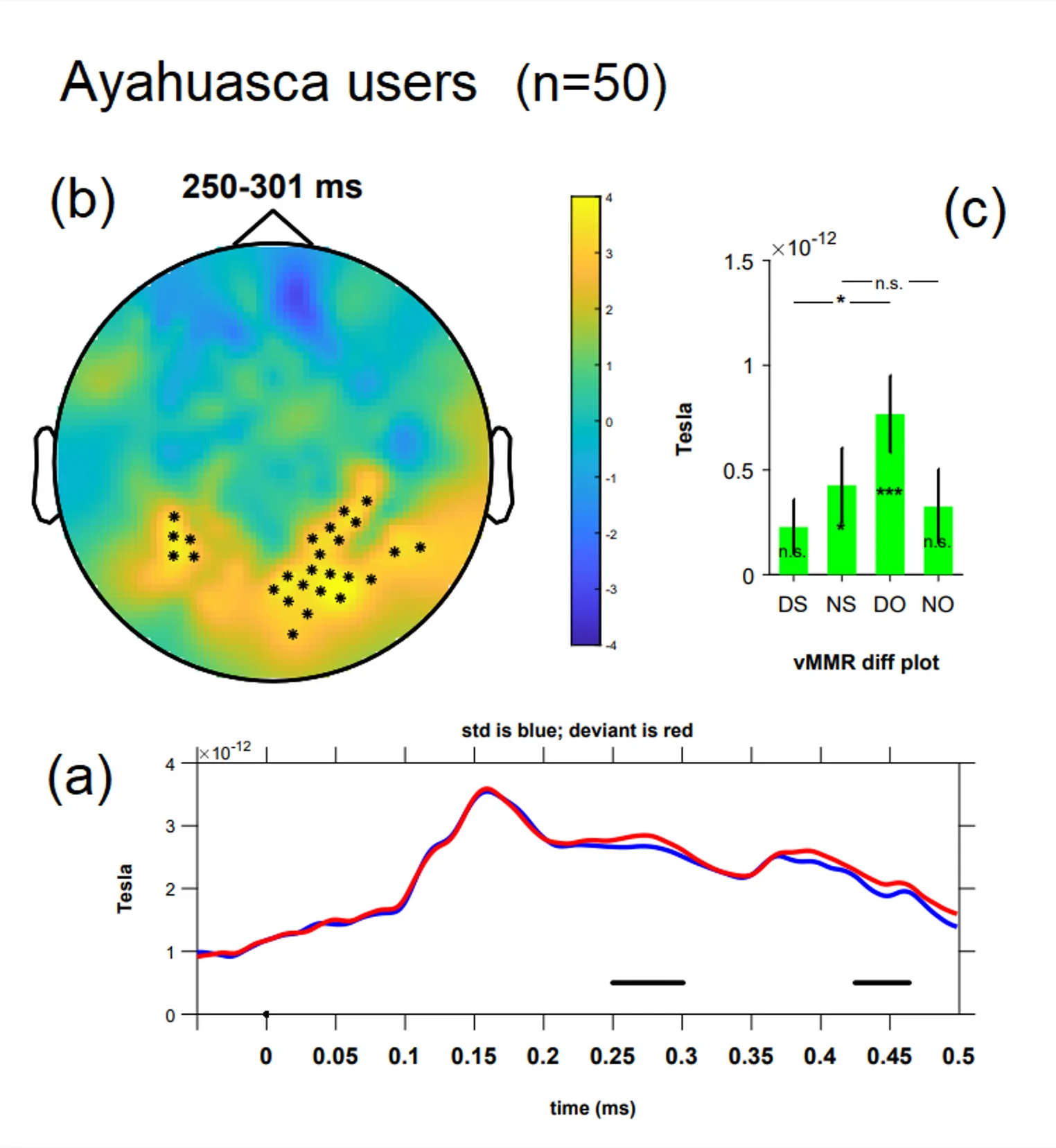
vMMR sensor-level results. (**a**) Event-related fields elicited by standards (blue) and deviants (red) averaged over all sensors. Significant time points (*p* <.05) are marked by black dots (250–301 ms post stimulus). (**b**) Topographical map of the deviants minus standards (*t-values)* averaged over the time window of significance. Sensors in the significant clusters are marked by bold stars. (**c**) vMMR difference plot (deviant minus standard) per condition averaged over the left- and right-posterior clusters. Error bars denote standard error of the mean. *=*p* <.05, **=*p* <.01, ***=*p* <.001. Abbreviations: vMMR = visual mismatch response; DS = death_self; NS = negative_self; DO = death_other; NO = negative_other; std = standard trials (in blue); dev = deviant trials (in red)

Following the methodology laid out in (Dor-Ziderman et al. 2019, 2025), cluster-based permutation statistics yielded two significant deviant > standard time-windows (Fig. 3a). The first was between **250 and 301** ms post-stimulus (*p* =.027), in line with the time-windows found in the previous studies (Fig. 2). The results were expressed in sensor space (Fig. 3b) as posterior-left (5 sensors, *p* =.016) and posterior-right (21 sensors, *p* =.002) clusters, replicating previous results regarding the left cluster (see Fig. 2a), and aligned with a right-posterior activation which was present in the original study but which did not reach significance (Fig. 2a), possibly due to its smaller sample size.

After collapsing the vMMR effect of the first time-window (250–301 ms) into the different conditions, a repeated-measures 2 × 3 ANOVA was computed with cluster LOCATION (left-/right-posterior), IDENTITY (self/other), and PRIME (death/negative) as within-subject factors. Main effects were not significant (*p*s > 0.18). Additionally, all the double and triple interactions with LOCATION were not significant (*p*s > 0.05). However, the IDENTITY and PRIME interaction was significant (F(1,49) = 8.05, *p* =.0007, $\eta_p^2$ = 0.141), indicating an effect beyond cluster lateralization. Post hoc analyses on the combined left and right cluster values by condition revealed a pattern which did not support our main hypothesis. The results indicated that unlike the meditators, and similar to the original sample (Fig. 2a), ayahuasca long-term users did not evidence a significant vMMR for the DS condition (*t* = 1.33, *p* =.191, *d* = 0.19, 95% CI [−0.09 0.47], *n.s*). However significant vMMR results were found for the DO (death but not self, *t* = 4.03, *p* <.001 [*p*_*HB*_<0.004], *d* = 0.57, 95% CI [0.27 0.87], Fig. 3c) and NS (self but death, *t* = 3.23, *p* =.002 [*p*_*HB*_=0.006], *d* = 0.46, 95% CI [0.16 0.75], Fig. 3c) conditions, thus verifying that the lack of results in the DS condition was no due to death or self features per se, but rather uniquely to their combination. Importantly, these effects could not be attributed to ayahuasca-related factors (detailed in SI2, SI Table 2). None of the consumptions metrics (lifetime usage, time of first use, time since last and most intense ceremony) nor (typical or most intense) ego dissolution values were associated with the DSD (all *p*s > 0.27, *n.s.*). Neither could these effects be accounted for by factors relating to lack of attention to facial stimuli in general, or dependent on condition. Repeated-measures ANOVAs on hit rates and RTs of target trials (pressing a button when a face with sunglasses appeared) verified that participants were attentive to stimuli (> 96% hit rate on all conditions), and that there were no differences (main effects or interactions) between conditions for hit rates and RTs (*p*s > 0.05). Finally, the word death-related and merely-negative word lists were balanced for valence, arousal, length, and frequency in the language (see SI1 and SI_Table 1), thus ruling out these serving as possible explanations for the vMMR results.

A later second time-window was detected between 424 and 464 ms post-stimulus (*p* =.042). It was expressed as a left-posterior cluster, which was only marginally significant (3 sensors, *p* =.054), and is thus not further discussed in the main text. However, see SI3 and SI Fig. 1 for further conditions-resolved analyses suggesting that for ayahuasca long-term users, the indices of death denial were present also at this later latency. In comparison, meditators also evidenced a later time-window, expressed as a large posterior bilateral cluster. However, this cluster was insensitive to condition (see SI2 and SI Fig. 1 in (Dor-Ziderman et al. 2025).

### Death processing in ayahuasca long-term users, experienced meditators, and general population

To formally quantify the vMMR differences between the ayahuasca long-term users relative to the previously published data of meditators and general population, we conducted an exploratory analysis by re-analyzing data already published (summarized in Fig. 2) on the original sample (controls) (Dor-Ziderman et al. 2019) and meditators (Dor-Ziderman et al. 2025). Importantly, data from all the groups were collected in the same lab using the same delivery setup for the stimuli and the same MEG apparatus for measuring brain activity. Furthermore, we showed (see methods) that the number of trials by condition did not differ between the present and previous groups.

Furthermore, the three groups (ayahuasca/meditators/controls) were compared over the left-posterior cluster which was present for all three groups (a parallel analysis including also the right-posterior cluster for the ayahuasca group yielded similar results). The time and space-resolved cluster-data was normalized (by subtracting the mean and computing z-scores), and a mixed ANOVA was computed with GROUP (ayahuasca/meditators/controls) as a between-subjects factor, and IDENTITY (self/other) and PRIME (death/negative) as within-subject factors. This manipulation allowed us to control for global signal measurement differences while maintaining the distribution pattern of the conditions’ for each group. Thus, while these group comparisons are inherently limited, they provide a means of formalizing the striking qualitative differences observed between the datasets (i.e., the DS vMMR being the largest in meditators but smallest in the other groups).

**Fig. 4:**
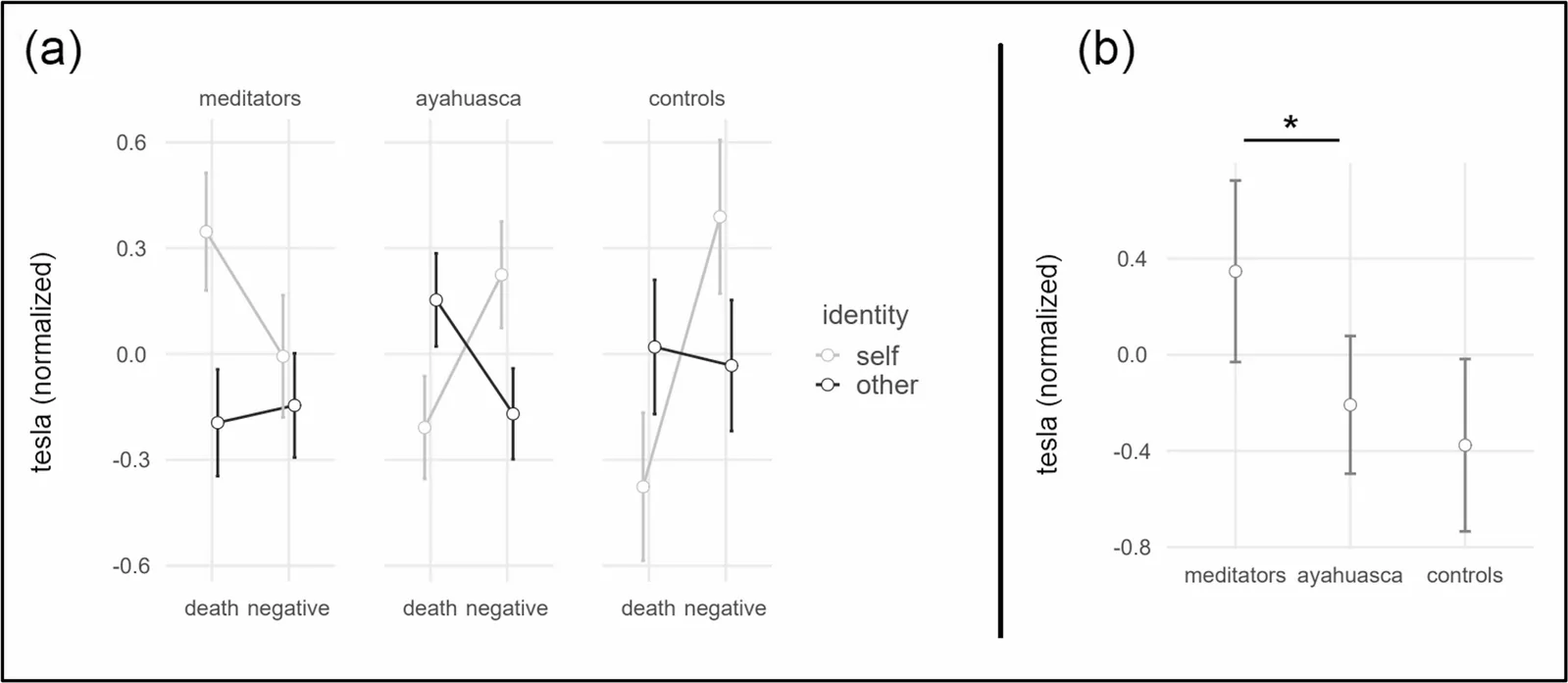
Self-specific group differences. (**a**) visual depiction of normalized vMMR by condition results highlighting the IDENTITY (self/other) X PRIME (death/negative) X Group (meditators/ayahuasca/controls) interaction. Error bars denote standard errors of the mean. Note the similarity of the ayahuasca pattern of results to the control group, both of which differ from the meditators pattern. The underlying differences were shown (see results) to be specific to the death-self (DS) condition, where (**b**) meditators evidenced higher normalized vMMR DS values than ayahuasca, which was not different from controls. Error bars denote 95% CI. *=*p* <.05

The results indicated a significant GROUP by IDENTITY by PRIME interaction effect (F(2,109) = 5.306, *p* =.006, $\eta_p^2$ = 0.089). As can be seen in Fig. 4a, the patterns of results were (descriptively) similar for ayahuasca long-term users and controls, but different for meditators. Mixed ANOVAs conducted for 2-group combinations verified this observation. GROUP by IDENTITY by PRIME interaction effects were seen for ayahuasca long-term users relative to meditators (F(1,86) = 8.554, *p* =.004 [*p*_*HB*_=0.008], $\eta_p^2$ = 0.09), but not relative to controls (F(1,72) = 0.019, *p* =.89, *n.s*). Finally, 1-way ANOVAs conducted for each of the four experimental conditions separately across groups showed a significant effect only for the DS condition (F(2,109) = 4.64, *p* =.012 [*p*_*HB*_=0.048], $\eta_p^2$ = 0.078), but not for the other three conditions (*p*s > 0.2). Post hoc analyses (Fig. 4b) showed lower DS values for ayahuasca than meditators (*t* = 2.51, *p* =.013 [*p*_*HB*_=0.026], *d* = 0.54, 95% CI [0.11 0.99]), but not different from controls (*t* = 0.66, *p* =.512, *n.s*). Overall, these results provide strong evidence regarding the self-specificity of the death denial effect, which was also the critical factor varying across groups.

**Fig. 5:**
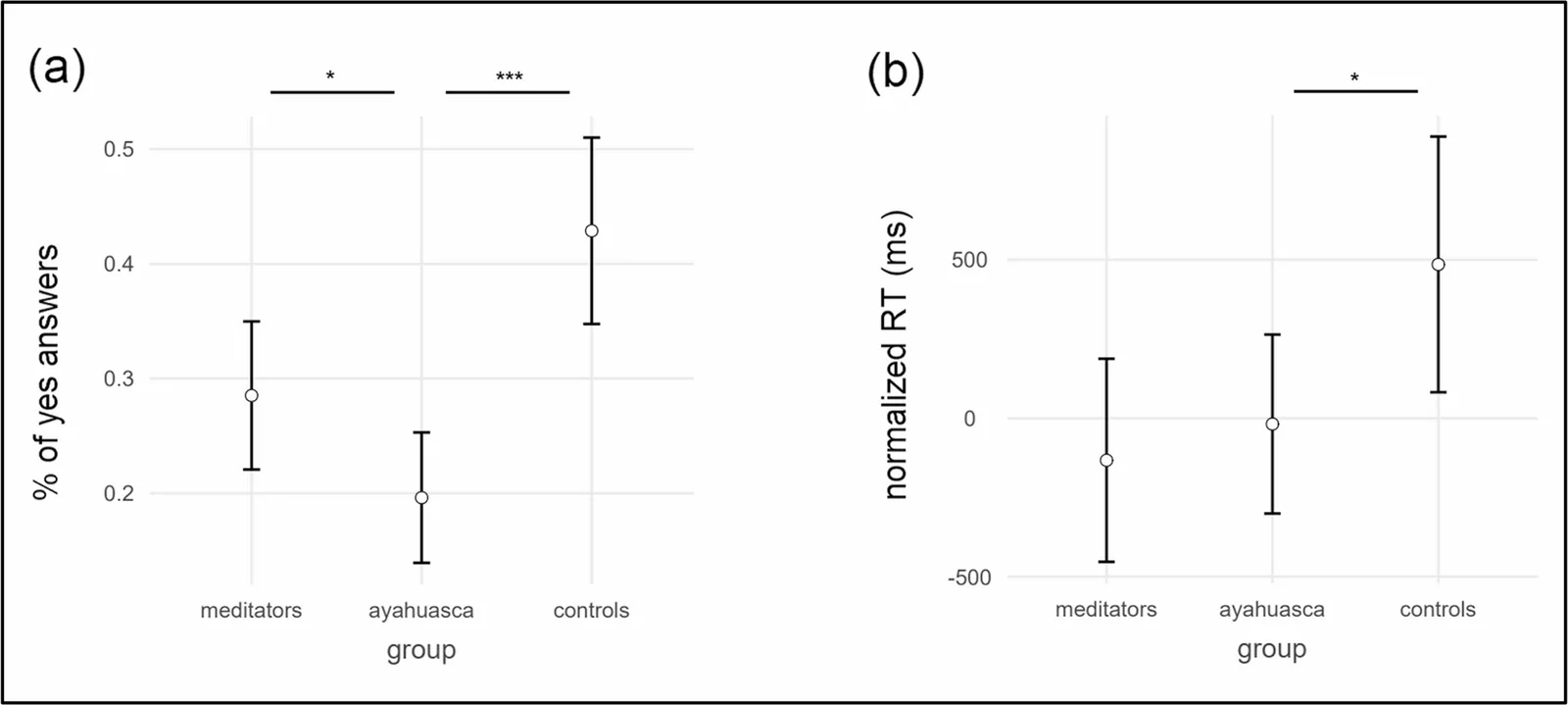
fear of death group differences between ayahuasca, meditators and controls shown for (**a**) the self-reported percentage of yes answer to death-related questions, and for (**b**) normalized (see methods) response latencies to the death-related items. Error bars denote 95% CI. *=*p* <.05, **=*p* <.01, ***=*p* <.001

It is important to note that despite clear evidence for death denial on fast and automatic level of the vMMR neural activity, results from the FPDS task which involved reflecting and answering questions, did point to a reduction in fear of death in ayahuasca group relative to the control sample. 1-way ANOVAs yielded a significant effect for percentage of yes responses to items (F(2,108) = 10.9, *p* <.001, $\eta_p^2$ = 0.17), as well as a marginally significant effect for response latencies (F(2,108) = 3.04, *p* =.052, $\eta_p^2$ = 0.053). Post hoc analyses showed that relative to controls, ayahuasca long-term users reported being less afraid of death (*t*=−4.65, *p* <.001, *d*=−1.16, 95% CI [−1.68 − 0.64], Fig. 5a), and evidenced faster responses (*t*=−2.03, *p* =.045, *d*=−0.51, 95% CI [−1 − 0.01], Fig. 5b) indexing as less implicit fear of death. Furthermore, the ayahuasca long-term users even reported less fear of death than the meditators (*t*=−2.05, *p* =.043, *d*=−0.44, 95% CI [−0.88 − 0.01], Fig. 5a), but were not different than meditators in their response latencies (*t* = 0.53, *p* =.597, *n.s*). Summary metrics on FPDS response distributions, including mean and standard deviation, can be found in the supplementary information section SI5, SI Table 3. Thus, a clear dissociation between levels of cognitive processing can be observed regarding death processing: indications of reduced fear of death at the level of reflection and behavior, but intact death denial indices at the level of fast, automatic and unconscious neurophysiological mechanisms.

### Preliminary evidence for construct validity and adaptive value of the vMMR death denial paradigm

As mentioned, we capitalized on the relatively large ayahuasca sample size to explore correlations between the DSD and several related constructs, with the aim of supporting the vMMR paradigm’s construct validity, as well as generally advancing a more complete understanding of its impact on everyday living experience. That is, its association with well-being and life satisfaction, and whether links existed between this unconscious denial mechanism and consciously experienced mortality-related affect.

**Fig. 6:**
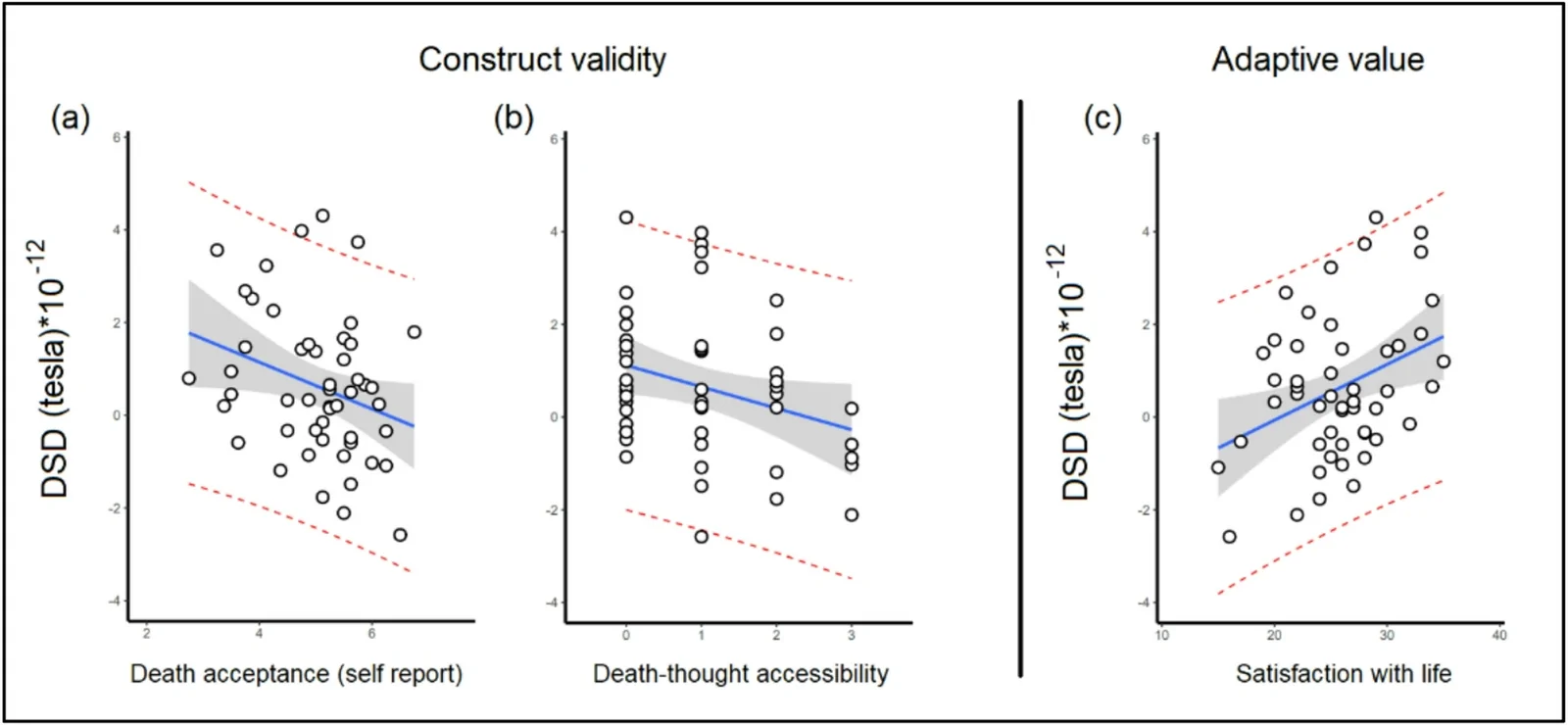
DSD correlations with denial-related measures including (**a**) self reported death acceptance and (**b**) death-related word completions in the death-thought accessibility task; as well as with (**c**) the satisfaction with life self report measure of flourishing

As can be seen in Table 2, correlation analyses did not provide support for the link between fear and anxiety of death and the neurophysiology of death denial (all *p*s > 0.05). In terms of construct validity, both tested measures yielded significant (LAPR-DA, *p* =.03) or marginally significant (DTA, *p* =.053) correlations. Larger neurophysiological values of death denial predicted less self-reported death acceptance (Fig. 6a), as well as less suppression of death-related thoughts (Fig. 6b), thus supporting the construct validity of the DSD. Finally, while the DSD did not predict psychological well-being (*p* >.05), it did predict perceived satisfaction with life (*p* =.01, Fig. 6c), thus indicating that for the ayahuasca long-term users, death denial may serve as an adaptive value. It is important to note that as none of these correlations results survive multiple comparisons corrections, they should be regarded as exploratory preliminary results which require further confirmatory evidence.

## Discussion

The current study examined the validity and scope of scientific and popular claims concerning the capacity of psychedelics to effect a fundamental transformation in the human relationship to death—specifically, by reducing denial, fear, and anxiety, and fostering greater acceptance of one’s mortality. By administering a previously-validated no-report predictive processing based vMMR MEG task to a population of experienced ayahuasca users, we were able to demonstrate that, while reducing anxiety and fear of death, contrary to our hypothesis, ayahuasca long-term users’ neurophysiological data displayed a pattern of results consistent with prior findings interpreted as markers of death denial, which in the context of the vMMR paradigm refers to an unconscious automatic categorization of death-related (but not merely-negative) stimuli as pertaining to ‘other’ (non-self/world), but not ‘self’(Dor-Ziderman et al. 2019).

Secondary analyses of previously published data were pivotal in demonstrating group differences as well as pinpointing the conditions under which these manifested. The ayahuasca long-term users’ pattern of results were similar to the original general population sample (Dor-Ziderman et al. 2019), but different from a subsequently collected meditators sample, which displayed neurophysiological markers of death acceptance (Dor-Ziderman et al. 2025). On the one hand, these results reinforce the rationale driving the death denial paradigm: that it is the combination of death and self-related stimuli which causes the human mind-brain to undertake protective action, and that at least on some level, death denial is a fundamental human phenomenon (Pyszczynski et al. 2015; Dor-Ziderman et al. 2019; Dor-Ziderman and Keenan 2024). On the other hand, these results, coupled with self-report and behavioral results indicating reduced fear of death in both the ayahuasca and meditators samples relative to the general population sample, suggest a more nuanced view than is often portrayed regarding the impact of psychedelics on death processing. In line with the general psychedelic literature (Davis et al. 2020; Gasser et al. 2015; Griffiths et al. 2011, 2016, 2018, 2019; Moreton et al. 2024; Moreton et al. 2023a, b; Sweeney et al. 2022; Yaden et al. 2017), ayahuasca does seem to lessen consciously experienced fear and anxiety of death. However, contrary to how ayahuasca and other psychedelics are often portrayed, their potential for fundamentally altering the human relationship with death may be limited. Earlier, faster, and non-conscious death denial processes seem to be unaffected by repeated naturalistic ayahuasca intake. Thus, the long-term impact of ayahuasca, and possibly other psychedelics, on death processing systems may be modulated by level of cognitive processing, affecting higher but not lower levels, and specifically not impacting processes hinging on self-specific processing (Christoff et al. 2011).

These secondary analyses were also important as they allowed pinpointing the conditions under which group differences manifested. The data clearly showed that differences between the ayahuasca long-term users and the meditators were specific to the condition coupling death and self (DS condition). No group differences were observed in the other three conditions (DO, NS, NO conditions). This finding lends further support to the fundamental premise underlying the vMMR paradigm: that the human brain is not aversive to death per se, but rather to death as it pertains to its own biological system (here operationalized using self facial images), ‘shielding’ the self model from information which could undermine its coherency (Metzinger 2024).

Further analyses supported the construct validity of the vMMR paradigm’s death denial construct. The DSD showed a significant, but modest, inverse correlation with a self-report measure of death acceptance, such that stronger neurophysiological denial markers predicted less self-reported acceptance of one’s mortality. Likewise, the DSD displayed a significant, but modest, inverse correlation with a behavioral measure of death-thoughts suppression, linking denial at the level of perception to denial at the level of thoughts. The associations between these different measures provide preliminary evidence that the constructs they underpin are related; however, their modest effect sizes indicate that they are distinct constructs. We also examined potential links between neurophysiological death denial mechanisms as a moderating factor down-regulating affective fear and anxiety responses to mortality. This notion was not supported as no correlations were observed between the DSD and the self-reported as well as behavioral measures of fear and anxiety of death. This is not to say that links between defensive responses and death anxiety are unsupported when examined through other experimental means. Reviewing the TMT literature, numerous behavioral studies (reviewed in (Juhl and Routledge 2016) demonstrate that experimentally heightening death awareness increases anxiety and decreases well-being for individuals who lack appropriate psychological buffers. However, we are not aware of any reports of trait, non-experimentally-induced linear correlations between death anxiety and mortality awareness. Thus, it is possible that such links exist but are not linear, or possibly that these effects are small and require a more targeted experimental manipulation. This is an important topic for future work. Likewise, our findings did not support a linear associations between well-being and death denial; however, we did find a correlation between one’s global assessment of life satisfaction and the DSD – thus lending initial support to the hypothesized adaptive coping value of mortality defenses (Pyszczynski et al. 2015; Qirko 2017; Varki 2019), that is, the modulation of death awareness for keeping at bay existential terror which can potentially disrupt the mundane imperatives of personal and societal life.

For the remainder of the discussion, we attempt to synthesize the results from the present and previous data sets, as well as relevant literature, to explain why ayahuasca long-term users retain intact neurophysiological markers of death denial. While a definitive account awaits future research, the multiple datasets and study variables allow advancing the field by ruling out several candidate explanations. These will be, in turn, presented (in italicized formatting) and discarded. Finally, a preliminary explanation will be offered.

* 1 st candidate explanation*: *The neurophysiological mechanisms of death denial are ‘hardwired’*,* that is*,* they cannot be changed. Thus*,* all attempts to confront the reality of mortality must work around these ingrained processes*. This explanation cannot be true as indicated by the meditators’ data which establishes that these processes are amenable to change.

*2nd candidate explanation*: *Meditators’ brains display neurophysiological acceptance of death due to buffering afterlife beliefs which blunt the harsh reality of death and conceptualize it as continuation rather than annihilation. Possibly the ayahuasca users do not hold such beliefs.* One argument is that ontological beliefs operating as part of a high-level cognitive self-model alter early low-level denial processes either directly or indirectly by enabling positively-valenced experiences (Thomas Metzinger, personal communication). While indeed the meditators displayed high certainty (median of 80% on the ABC scale which ranges from − 100 to 100, see methods section) in the continuation of consciousness (Dor-Ziderman et al. 2025), this was equally true for the ayahuasca participants who displayed a median of 90% certainty (SI Table 2) in the continuation of consciousness after death. More generally, psychedelic consumption has been associated with non-materialistic views of consciousness (Timmermann et al. 2021), as well as beliefs such as reincarnation, communication with the dead, and existence after death (Nayak et al. 2023).

*3rd candidate explanation*: *Altering neural death denial processes requires experiences where the boundaries between self and other dissolve (as suggested by the meditators’ dataset). Possibly the participants in our sample did not have such experiences*. Generally, psychedelics are associated with self/ego-dissolution experiences which are characterized by dissolution of self-world boundaries (Nour et al. 2016; Letheby and Gerrans 2017; Millière et al. 2018), and its neural basis (Lebedev et al. 2015; Tagliazucchi et al. 2016; Smigielski et al. 2020). Specifically in the present ayahuasca sample, participants’ EDI scores (ranging from 0 to 100, see methods and SI Table 2) indicated that they had experienced strong (mean = 82.7; only 2 under 50) ego dissolution, with typical ego-dissolution ceremony experiences also being high (mean = 62.7).

*4th candidate explanation*: *Psychedelics do not alter prediction-based self-specific MMR neurophysiological processes during acute experiences*,* thus there is no reason to suspect parallel long-term changes*. There are mixed findings regarding alterations to basic sensory predictive processing mechanisms under acute psychedelics’ effect (Umbricht et al. 2003; Heekeren et al. 2008; Schmidt et al. 2012; Timmermann et al. 2018; Duerler et al. 2022). Two EEG studies investigating psilocybin (Schmidt et al. 2012; Umbricht et al. 2003) and one study on DMT (Heekeren et al. 2008) failed to demonstrate the effect of psychedelics on acute auditory MMN. However, more recent, better designed, and more highly powered studies indicated otherwise. Timmermann et al. (2018) demonstrated that LSD reduces the magnitude of the MEG MMR response in an auditory oddball paradigm, and Duerler et al. (2022) showed that psilocybin reduced tactile MMR EEG responses at frontal electrodes. Thus, it appears that psychedelics do impact basic sensory processes under acute conditions, however, the final verdict is yet to be determined. However, and directly relevant to the present study, there is evidence of acute psychedelics effects on self-specific MMR ERP components. An intriguing study by Smigielski and colleagues (Smigielski et al. 2020) showed that psilocybin abolished the distinctiveness of self- and other-related electric field configurations during the P300 timeframe in an auditory self-other task. In a similar vein, and with even greater relevance to the present study, a recent study (Suay et al. 2024) found that an ayahuasca-inspired DMT-Harmine formulation significantly blurred the P300 neural distinction between self and other faces under acute conditions.

In summary, the intact vMMR death denial mechanisms in ayahuasca long-term users cannot be explained away as processes which are ‘hardwired’, as processes which are not impacted under acute conditions, or that require certain ontological beliefs as pre-conditions for change. Furthermore, the ayahuasca participants, like the meditators (Dor-Ziderman et al. 2025), had undergone powerful self/ego-dissolution experiences which blur self-other boundaries, so this cannot be the differentiating factor. Thus, we propose that it may be the case that self/ego-dissolution acute experiences alone are not sufficient for generating associated long-terms effects in embodied self-specific processes. Given that the vMMR death denial mechanisms operate at a perceptual level differentiating self from non-self, their alteration as long-terms effects may be contingent upon long-term effects to self-specific embodied processes. However, such changes may require developing and enhancing attentional capacities. Meditation is first and foremost a training of one’s attention. Numerous studies on meditation have established that meditation training enhances attentional processes and their associated neural mechanisms in a trait-like manner (Tang et al. 2015; Goleman and Davidson 2017; Sumantry and Stewart 2021), and is coherent with predictive processing accounts (Lutz et al. 2019; Laukkonen and Slagter 2021). Meditation practice has also been shown to induce strong changes in the acute sense of self and its neural basis (Dor-Ziderman et al. 2013, 2016; Ataria et al. 2015; Nave et al. 2021; Trautwein et al. 2024). Finally, and of particular interest, is the study by (Trautwein et al. 2016) who assessed the long-term P300 event-related potential elicited by oddball stimuli of the self-face relative to a close other’s face in practitioners of Loving-Kindness Meditation and matched controls. The study found reduced P300 self-prioritization effects in the meditation sample compared to controls indicating altered self-processing in meditators in a trait-like manner. Furthermore, meditation practice degree predicted P300 self-bias magnitude.

In comparison, there is no rigorous evidence showing long-terms attentional effects in psychedelic populations (Aday et al. 2020). Reviewing the literature, existing evidence is limited to a cross-sectional study (Bouso et al. 2012) attributing enhanced performance in attentional tasks to a sample of regular ayahuasca users in a ritualistic, religious context (Santo Daime church), as well as a more recent study reporting unexpected results of altered (but not necessarily enhanced) attentional neuropsychological P300 ERP components (Orłowski et al. 2024). Crucially, this last study attempted to replicate the auditory P300 self-specific acute effects mentioned above (Smigielski et al. 2020) on experienced psychedelic users but not under psychoactive influence. However, no effects were found. The authors concluded that while psychedelic experiences have been reliably associated with a blurring of the self boundaries (Nour et al. 2016; Letheby and Gerrans 2017; Millière et al. 2018; Smigielski et al. 2020), repeated psychedelics usage in naturalistic contexts may not result in more permanent alterations in perceptual processing of self-specific stimuli (but possibly yes in higher-order narrative reflective aspects of self-consciousness (Orłowski et al. 2022) which have received more attention in the literature (Girn and Christoff 2018). Thus, the factor differentiating experienced meditation practitioners from long-term ayahuasca users in terms of self-specific acceptance or denial processes may not be self/ego-dissolution experiences per se, but rather their underlying causal mechanism: attentional training for the former, and psychopharmacological modulation for the latter. This notion is a hypothesis which should be tested in future studies.

Viewed from a hierarchical predictive processing perspective (Friston 2010; Hohwy 2013), long-term meditation-induced attentional skills may recalibrate precision-weighting within generative self-models (Lutz et al. 2019; Laukkonen and Slagter 2021), attenuating rigid high-level priors that normally down-weigh death–self prediction errors. Such recalibration aligns with evidence of altered default mode network dynamics—reduced dominance of narrative-self hubs and increased coupling with attentional networks (Brewer et al. 2011; Garrison et al. 2015)—and may facilitate a shift from narrative to embodied self-representations (Christoff et al. 2011; Dor-Ziderman et al. 2013; Metzinger 2024). This framework accounts for meditators’ robust mismatch responses to death–self pairings and contrasts with ayahuasca long-term users, whose persistent suppression of this effect may suggest unaltered, deeply embedded lower-level priors sustaining self-specific generative models and their associated mortality-denial mechanisms. Nevertheless, in line with the REBUS model of psychedelic action (Carhart-Harris and Friston 2019) and observed DMN attenuation (Carhart-Harris et al. 2012; Palhano-Fontes et al. 2015), psychedelics may temporarily relax higher-order priors allowing acute self/ego-dissolution experiences to arise, which are, in turn, may provide “evidence” for narrative trajectories in which some version of the self persists after death, thereby modifying aspects of narrative self-representation without restructuring embodied self-models.

As a final note, combining psychedelics with intense meditation practice may be highly effective. An intriguing study (Smigielski et al. 2019a), in which psilocybin (with placebo controls) was administered to experienced Zen meditators in naturalistic retreat setting, found that psilocybin increased meditation depth and incidence of positively experienced self-dissolution. It was also associated with the decoupling of medial prefrontal and posterior cingulate cortices within self referential networks (Smigielski et al. 2019b). Both the experiential and neural indices predicted enhanced psychosocial functioning at a 4-month follow-up. Thus, there is a case to be made for combining pharmacological and non-pharmacological elements for shaping the experiential quality and valence of psychedelic ‘selfless’ states, as putative modulators of behavior and attitudes, in general, and specifically as related to death processing.

The current study has several limitations, the most salient one being that the ayahuasca long-term users were not compared head-to-head with control or meditators groups, but rather relied on secondary analyses of previously published data. However, it is important to reiterate that while these samples were examined at different time periods, the same paradigm in the same MEG scanner using the same analyses procedures were employed. Furthermore, the case for the existence of group differences in the neurophysiological mechanisms of death denial were not based on differences in degree of reported effects, but rather on a qualitatively different pattern of the paradigm’s conditions. An additional important limitation of the study regards its cohort of highly experienced ayahuasca participants. It can be argued that long-term users represent a subgroup characterized by differential cognitive plasticity resulting from extensive ayahuasca usage. While this may be true, as the extent of lifetime ayahuasca intake has been associated with degree of lasting changes in brain structure (Bouso et al. 2015), we would expect enhanced rather than reduced cognitive plasticity in this population. Long-term ayahuasca users were shown to perform better on neuropsychological cognitive tasks under the acute effects of ayahuasca compared to occasional users (Bouso et al. 2013) – suggesting ayahuasca-induced enhanced cognitive plasticity. Another related concern is that the current sample suffers from a self-selection bias stemming from certain traits or positive experiences with ayahuasca contributing to continued use, while others who have certain experiences or negative experiences discontinue usage. Thus, it may be the case that naïve or short-term ayahuasca users might exhibit different results. While we cannot rule this out, our results, as well the literature, do not support lifetime usage degree predicting death processing metrics. Furthermore, ego dissolution experiences, which are more likely to be experienced with continued usage, have been associated with reduced fear and anxiety of death (Sweeney et al. 2022; Garcia et al. 2025), and are thus more likely to also enhance qualities such as death acceptance. Nevertheless, to rigorously and definitively counter these limitations, future studies should more rigorously compare psychedelic, meditative, and psychedelic/meditation-naive groups while controlling for potential baseline differences. This would be ideally achieved by employing a longitudinal design (rather than a cross-sectional), where naïve participants would be randomized to active control, psychedelic, and meditative interventions. Finally, the novelty of the FPDS_RT measure constrains its current interpretability, necessitating future research to rigorously evaluate its conceptual underpinnings and psychometric validity.

Despite these limitations, the present study makes a significant and innovative contribution to both the existential and psychedelic fields by pointing out the promise and limitations of psychedelics in transforming how humans perceive and conceive of the notion of their finitude. Better understanding the unconscious neural processes underlying how humans approach their mortality, their relation to felt anxiety and fear of death, and their possible clinical implications, may be instrumental for future development and assessment of interventions and practices aimed at inculcating a saner and more accepting approach towards the inevitable in populations faced with terminal diagnoses, but also in the general population, which faces a perhaps delayed, but no less certain, outcome.

## Supplementary Information

Below is the link to the electronic supplementary material.

Supplementary Material 1

## Data Availability

Data and code will be shared on request to the corresponding author after signing a data sharing agreement and contingent on approval from the requesting researcher’s local ethics committee.

## Declarations

We confirm that this work is original, has not been published elsewhere, and is not currently under consideration for publication elsewhere. The study has been, however, uploaded as a preprint to an OSF repository (https://osf.io/preprints/osf/my9sd_v1)
