# Supplementary material for: Intact neurophysiological markers of death denial in long-term ayahuasca users
Source: Psychopharmacology (Berl). 2025 Nov 10;243(9):2165–83. doi: 10.1007/s00213-025-06963-z (PMC13601162; doi:10.1007/s00213-025-06963-z)
Supplement: Supplementary file 1 [file 213_2025_6963_MOESM1_ESM.docx]

**Supplementary Information (SI)**

***SI1: vMMR task Stimuli and procedure***

The vMMR task’s facial stimuli, word stimuli, and procedure were similar to that employed in our previous publication (Dor-Ziderman et al., 2019). These are described briefly below.

Face Stimuli consisted of Six grey-scaled pictures of faces (425 x 405 pixels) with neutral expressions were presented centrally on a black foreground, using E-prime2 software (Psychology Software Tools). Face images included the participant’s face (SELF), a gender-matched individual (OTHER) and a morphed face that contained 50% of the participant’s face and 50% of the gender-matched individual (MORPH). The morph images were created using FantaMorph4 (Abrosoft FanatMorph Software). In addition, target images were created by adding black sunglasses to the three images described above, yielding a total of six images per subject. All photographs were taken in a designated location against a white wall under similar lighting conditions. Non-facial features were masked out using a round black outline and the images were equalized for low level image properties (luminance, contrast, intensity and spatial frequency) using the SHINE toolbox (Willenbockel et al., 2010).

Word stimuli consisted of 72 words of two categories (36 words in each): death-related (DEATH) and negative (NEG). The words were selected from an existing database (Shay, 2016) using an in-house evolutionary algorithm designed to optimize word-selection so that the word categories would be (1) equivalent in terms of word frequency (in Hebrew, based on Google search), length (number of letters), and concreteness (subjective ratings), and that (2) the DEATH and NEG stimuli would also be equal in terms of valence and arousal ratings, but different in their degree of relation to death (on Likert 1-7 scales, ratings obtained from an independent population).

|  | **Valence** | **Arousal** | **Death-relatedness** | **Concreteness** | **Frequency** | **Length** |
| --- | --- | --- | --- | --- | --- | --- |
| **Death** | 6.38 (.6)  # | 5.3 (.82)  # | 6.32 (.83)  * | 4.25 (1.45)  # | 632.27 (874.44)  # | 4.67 (1.33)  # |
| **Negative** | 6.24 (.56)  # | 5.2 (.58)  # | 1.9 (.52)  * | 4.25 (1.65)  # | 629.56 (1019.65)  # | 4.66 (1.55)  # |
|  | Column cells sharing a # symbol are **not** significantly different (p > .1, BF <= 0.243)  Column cells sharing a * symbol are significantly different (p < .001, BF > 1000) | | | | | |

**SI Table 1.** **Word stimuli properties and ratings.** Information provided for each word category (death/negative) include median and interquartile range (IQR) values of Likert 1-7 scale ratings of valence, arousal, death-relatedness, and concreteness; as well as mean and *SD* of the words’ frequency (in Hebrew) and length (number of letters). Significance is computed using the Mann-Whitney U test and Bayes Factors (BF) for Likert-type scales variables, and for continuous variables using independent-samples t test and BF.

Procedure: We implemented a fully-randomized 2x2 design with PRIME (DEATH or NEGATIVE) and IDENTITY (SELF or OTHER) as within-subject factors. Each trial started with the presentation of a small fixation cross on the screen for 500-700 ms and then a prime word (DEATH or NEG) for 600 ms. After that, directly underneath the prime word, a central cross appeared for 250 ms and then a sequence of face images. Each sequence consisted of identical 3-6 standard (SELF or OTHER) face images and then a deviant face (MORPH). Each face was presented for 250 ms with a stimulus onset asynchrony of 600 ms. At any point during the sequence, a target face could appear matching the sequence type (SELF or OTHER) and sequence location (standard or deviant). Subjects were instructed to press a button with their index finger as soon as a target image was detected. Hit rates as well as response latencies were collected. Beyond maintenance of participants’ alertness and focus on the facial stimuli, these behavioral measures were included to rule out attention as a significant mediator. The experiment was divided into three blocks, and preceded by a practice block. Between each block participants were requested to close their eyes and rest. Each block consisted of 120 trials (prime word plus the faces sequence), 30 of which included targets and were not included in the ERF analysis. The task took around 35 minutes to complete. See Figure 1 (in the main text) for a visual depiction of the study setup.

***SI2: Demographics and ayahuasca related factors***

| **Demographics**  Age |  | 38.4±8.9 |
| --- | --- | --- |
| Gender | Male  Female | 33 (66%)  17 (34%) |
| Education | High School or equivalent | 9 (18%) |
|  | College Diploma/certification | 32 (66%) |
|  | Master’s Degree and above | 9 (18%) |
| Family status | Unmarried | 25 (50%) |
|  | Married | 16 (32%) |
|  | Divorced | 9 (18%) |
| Income | Below average | 12 (24%) |
|  | Average | 22 (44%) |
|  | Above average | 16 (32%) |
| Religion | Secular  Religious | 31 (62%)  0 (0%) |
|  | Traditional | 8 (16%) |
|  | Other | 11 (22%) |
| **Ontological belief** | ABC | 77±42.9 |
| **Ayahuasca** |  |  |
| consumption habits  ego-dissolution experiences | Lifetime use (number of times ayahuasca was taken) | 58.4±84.8 |
|  | Age of first ayahuasca | 31.3±8.4 |
|  | Last use (month) | 5.9±6.3 |
|  | Most intense (month) | 34.9±36.6 |
|  | EDI – strongest | 82.7±19.2 |
|  | EDI - typical | 66.7±21 |

**SI Table 2.** Sample **demographics** including age, gender, education, family status, and income; **ontological** After life Belief Certainty (ABC) scores (range: -10 to 10, certainty in annihilation to continuation) and **ayahuasca** consumption parameters including lifetime use, age of first usage, last time ayahuasca was consumed, time since most intense ceremony, and the Ego Dissolution Inventory (EDI) ratings (range: 0-100) for the ‘most intense’ and ‘typical’ past acute ego dissolution experiences.

***SI3: vMMR results for the 424-464 ms cluster***

As shown in SI figure 1a, a small left-posterior cluster was marginally significant (3 sensors, *p*=.054) at the late time-window (424-500 ms). The resulting vMMRs were collapsed over the cluster sensors and time-window into the different conditions. The results indicated significant vMMR effects in all the conditions (1-sample *t*-tests, *p*s<.05, SI figure 1b) except the DS condition (*t*=.02, *p*=.986, *n.s*). A repeated-measures 2x2 Anova with IDENTITY (self/other), and PRIME (death/negative) as within-subject factors, revealed a significant interaction effect (F(1,49)=4.785, *p*=.034, $\eta_{p}^{2}$=.09). Post hoc *t* tests revealed that the DS condition was significantly smaller than the DO condition (*t*=-2.9, *p*=.006, *d*=-.41, 95% CI [-.7 -.12]), mirroring the earlier effect described in the main text. The other comparisons were not significant (*p*s>.1).

**SI Figure 1:** **vMMR results for the 424-464 ms cluster.** (a) Topographical map of the deviants – standards (*t-values)* averaged over the time window of significance. Sensors in the significant cluster are marked by bold stars. (b) vMMR difference plots (deviant minus standard) per condition. Error bars denote standard error of the mean.

*=*p*<.05, ***p*<.01. Abbreviations: vMMR=visual mismatch response; DS=death_self; NS=negative_self; DO=death_other; NO=negative_other.

***SI4: vMMR lateralized results for the 250-301 ms cluster***

As described in the main text, the 250-301 time window yielded two significant clusters which were treated as one cluster as the ensuing mixed Anova interaction effect was not sensitive to LOCATION (left/right). However, as can be seen in SI figure 2, the left and right clusters yielded condition-resolved patterns which differ (descriptively) concerning the self enhancement effect under negative priming. The NS condition demonstrates a significant vMMR effect for left (*t*=3.65, *p*<.001, *d*=.52, 95% CI [.22 .81])) but not right (*t*=1.29, *p*=.205, *n.s*) cluster’s vMMRs. The left NS vMMR is also larger than the right NS vMMR (*t*=2.69, *p*=.01, *d*=.38, 95% CI [.09 .66]). And finally, as self enhancement effect (NS>NO) can be seen only in the left cluster (*t*=2.1, *p*=.041, *d*=.3, 95% CI [.01 .58]), but not in the right cluster (*t*=-.38, *p*=.702, *n.s*). This left lateralization is an interesting and novel effect which can be explored in future studies.

**SI Figure 2: vMMR lateralized results for the 250-301 ms cluster.** (a) Topographical map of the deviants – standards (*t-values)* averaged over the time window of significance. Sensors in the significant cluster are marked by bold stars. vMMR difference plots (deviant minus standard) per condition for the (b) left, and (c) right posterior clusters. Error bars denote standard error of the mean. *=*p*<.05, ***p*<.01, ****p*<.001. Abbreviations: vMMR=visual mismatch response; DS=death_self; NS=negative_self; DO=death_other; NO=negative_other.

***SI5: FPDS behavioral performance metrics***

| *Group* | *FPDS_RT* | *Dental pain_RT* |
| --- | --- | --- |
| **ayahuasca** | 4468±1674 | 4487±1273 |
| **meditators** | 3799±1205 | 3932±1838 |
| **controls** | 4384±1449 | 3898±900 |

**SI Table 3.** Summarizes the means and standard deviations of the implicit (reaction times in *ms*) data of the FPDS task, and its control dental pain condition for the ayahuasca, meditators, and controls groups.
